# Draft genome sequence of *Rhodococcus rhodochrous* IEGM 1360 isolated from Arctic soil and able to transform oleanolic and glycyrrhetinic acids

**DOI:** 10.1128/mra.00800-25

**Published:** 2025-08-25

**Authors:** Natalia A. Plotnitskaya, Irina B. Ivshina

**Affiliations:** 1 Perm Federal Research Center of the Ural Branch of the Russian Academy of Sciences 307499, Perm, Russia; 2 Perm State University 64949, Perm, Russia; Rochester Institute of Technology, Rochester, New York, USA

**Keywords:** *Rhodococcus rhodochrous*, oleanolic acid, glycyrrhetinic acid, Arctic soil

## Abstract

We report the draft genome sequence of *Rhodococcus rhodochrous* IEGM 1360. This strain was isolated from Franz Josef Land and can transform oleanolic and glycyrrhetinic acids, pentacyclic triterpenoids commonly used for obtaining pharmacologically significant compounds. Using the sequence, putative genes encoding enzymes involved in the transformation of triterpenoids were discovered.

## ANNOUNCEMENT

Oxidized derivatives of oleanolic and glycyrrhetinic acids exhibit diverse biological activities (1, 2). Microbial cells, particularly actinomycetes of the genus *Rhodococcus*, are increasingly used for biotransformation of a wide range of organic compounds (3–5) due to their high regio- and stereoselectivity in single-step reactions (6). These biocatalytic processes occur under mild conditions, use non-toxic media, and align with green chemistry principles by minimizing environmental impact (7).

The *R. rhodochrous* strain IEGM 1360, isolated from Arctic soil (Franz Josef Land, Arkhangelsk region, Russia) via the enrichment culture method, is deposited in the Regional Specialized Collection of Alkanotrophic Microorganisms (acronym IEGM, WFCC number 285, http://www.iegmcol.ru/strains/rhodoc/rhodoch/r_rhod1360.html). This strain can biotransform 1.0 g/L of oleanolic and glycyrrhetinic acids, yielding 61% and 100% of their respective 3-oxo derivatives (8). 3-Oxo-oleanolic acid exhibits anti-tumor, cytotoxic, antiparasitic, and anti-inflammatory activities (9–12), while 3-oxo-glycyrrhetic acid has anti-lipoxygenase activity (13).

DNA was extracted from cells grown in LB broth at 28°C and 160 rpm for 28 h using the Quick-DNA Fungal/Bacterial Microprep Kit/Quick-DNA Fecal/Soil Microbe Microprep kit (Zymo Research) following the manufacturer’s protocol. The extracted DNA was quantified using a Qubit 4 Fluorometer, and quality was assessed by electrophoresis in 1% agarose gel. A paired-end library (2  ×  100 nucleotides [nt]) was produced using Illumina DNA Prep and sequenced on an Illumina NovaSeq 6000. A total number of 19,807,415 reads was obtained. Demultiplexing of the sequenced reads was performed with Illumina bcl2fastq (2.20). Adapters were trimmed with Skewer (version 0.2.2) (14). The quality of the FASTQ files was analyzed with FastQC (version 0.11.5-cegat) (15). The data were assembled with SPAdes v. 3.14.1 (16) at the scaffold level. Assembly statistics were computed using QUAST 5.2.0 (17). The assembly consisted of 113 contigs, with a total sequence length of 5,731,318 bp, an N50 value of 203,477, a GC content of 68%, and coverage of 346×. The annotation of coding sequences (CDS) was performed using PGAP 5.3 (annotation method “best-placed reference protein set,” GeneMarkS-2+) (18) and RAST 2.0 (annotation scheme RASTtk) (19). Default parameters were used for all software, unless otherwise specified.

For taxonomic classification, average nucleotide identity (ANI) was calculated using the online tool (https://www.ezbiocloud.net/tools/ani) (20), and the highest OrthoANIu value of 97.89% was obtained for *R. rhodochrous* (NCBI: txid1829). Digital DNA-DNA hybridization (dDDH) was calculated using GGDC 4.0 (Genome-to-Genome Distance Calculator 4.0, https://ggdc.dsmz.de/ggdc.php#) (21, 22). The calculated dDDH values of 82.5%–85.8% with *R. rhodochrous* confirmed the taxonomic classification of strain IEGM 1360 as *R. rhodochrous*.

A total of 5,295 CDS, 5,215 CDS with protein, 5 rRNAs (1 5S, 1 16S, and 3 23S), and 54 tRNAs were found in the *R. rhodochrous* IEGM 1360 genome. Among the CDSs were 63 monooxygenases, 32 dioxygenases, 12 hydroxylases, seven peroxidases, and 348 dehydrogenases. Key monooxygenases included 10 cytochrome P450s (CYP450s), eight cyclohexanone monooxygenases, three 3-ketosteroid-9α-monooxygenases, and two steroid C27-monooxygenases. The CYP450 enzymes identified in the genome are likely involved in oxidative transformation of oleanolic and glycyrrhetinic acids, suggesting their potential role in the bioconversion of these triterpenoid compounds.

## Data Availability

This Whole Genome Shotgun project has been deposited in GenBank under the accession number JAJNCN000000000.1. Raw sequence reads were deposited in the Sequence Read Archive with accession number SRR17030450 under BioSample accession number SAMN23420026 and BioProject accession number PRJNA783162.
